# Supplementary material for: Toward Sensor-Based Sleep Monitoring with Electrodermal Activity Measures
Source: Sensors (Basel). 2019 Mar 22;19(6):1417. doi: 10.3390/s19061417 (PMC6470539; doi:10.3390/s19061417)
Supplement: Supplementary file 1 [file sensors-19-01417-s001.pdf]

| ***Question*** | ***Question Description*** |
| --- | --- |
| 1 | What time did you go to bed? (e.g. 11:30 pm) |
| 2 | How long, in minutes, did it take you to fall asleep? |
| 3 | What time did you get up this morning? (e.g. 9:00 am) |
| 4A | How many hours of sleep did you get last night? (e.g. 8) |
| 4B | How many hours were you in bed? (e.g. 9.5) |
| 5 | Did you take any medicine, prescribed or over the counter, to help you sleep? |
| 6 | Yesterday, did you have trouble staying awake while driving, eating meals, or engaging in social activity? |
| 7 | Yesterday, how much of a problem was it for you to keep up enthusiasm to get things done? |
| 8 | Last night, how would you rate your overall sleep quality? (1=Very Poor, 2=Fairly Poor, 3=Fairly Good, 4=Very Good) |
| 9 | Please list any occurrences that you can recall from last night, including time when they happened. Examples include: felt cold, felt hot, had pain, coughed or snored loudly, uncomfortable breathing, etc. |
